# FOXM1-induced TYMS upregulation promotes the progression of hepatocellular carcinoma

**DOI:** 10.1186/s12935-021-02372-2

**Published:** 2022-01-29

**Authors:** Liang Wang, Caiyan Shi, Jie Yu, Yilin Xu

**Affiliations:** 1 The Department of Radiation Oncology, Hainan Cancer Hospital, Haikou, 570311 Hainan China; 2 Department of Nephrology, Affiliated Huaian Hospital of Xuzhou Medical University, Huaian, 223001 Jiangsu China; 3 Department of Anal-Colorectal, Jingzhou Central Hospital, The Second Clinical Medical College, Yangtze University, Jingzhou, 434020 Hubei China; 4 The Department of Medical Oncology, Hainan West Central Hospital, Danzhou, 571700 Hainan China

**Keywords:** TYMS, FOXM1, Hepatocellular carcinoma, 5-FU resistance

## Abstract

**Background:**

Hepatocellular carcinoma (HCC) is the most common type of primary liver cancer and one of the major causes of cancer-related death. Thymidylate synthase (TYMS) catalyzes the methylation of deoxy guanosine to deoxy thymidylate, which is a crucial gene for DNA repair and replication. Thus, TYMS was reported to be closely associated with developing a variety of tumors, but it has been poorly studied in HCC.

**Materials and methods:**

We used the cell counting kit-8 (CCK-8), BrdU, and CFSE assay to measure cell proliferation. The flow cytometry assay and the TUNEL assay were used for assessing cell apoptosis. The flow cytometry assay was used to analyze the cell cycle. The Transwell invasion assay and the wound healing assay were conducted to determine the invasive ability of the cells. RT-qPCR and Western blot analyses were performed to evaluate the mRNA and protein expression levels of specific genes, respectively.

**Results:**

TYMS was found to be upregulated in both HCC cells and patient samples. High expression of TYMS was associated with an unfavorable prognosis in HCC patients based on the TCGA-LIHC dataset. Cell proliferation, apoptosis, and invasion assays revealed that TYMS promoted the proliferation and invasion of HCC cells as well as inhibited apoptosis. In addition, TYMS is a downstream target of FOXM1. TYMS knockdown reversed the 5-FU resistance caused by FOXM1 overexpression and re-sensitized HCC cells to 5-FU treatment.

**Conclusion:**

This study suggested that TYMS serves as an oncogene in HCC, and targeting the FOXM1-TYMS axis may help improve the survival of HCC patients as well as provide new insights for treating advanced HCC patients.

**Supplementary Information:**

The online version contains supplementary material available at 10.1186/s12935-021-02372-2.

## Introduction

Hepatocellular carcinoma (HCC) is the second most deadly cancer after pancreatic cancer [1]. In addition, HCC is the sixth most common cancer among all types of tumors. HCC usually occurs in patients with underlying liver diseases, including viral hepatitis (hepatitis B or hepatitis C), alcoholic hepatitis, and nonalcoholic fatty liver disease [2–6]. The prognosis of early HCC is excellent, and the average survival time is greater than 5 years. However, an advanced or terminal stage HCC prognosis has an average survival time of 8–13 months or 3 months, respectively [7]. HCC usually does not exert noticeable symptoms in its early stages and is mainly detected through a physical examination. Moreover, HCC is often found in the advanced stage in less medically developed areas. Sorafenib is the primary first-line treatment for advanced-stage HCC since 2007 [8]. Even though sorafenib is widely used, its effects vary broadly among patients of different ethnicities. Studies have shown that it works more optimally in Western populations than in Asians, not inducing complete remission [9]. Therefore, FOLFOX4 (oxaliplatin, folinic acid, and bolus fluorouracil (5-FU)) is widely used as a standard systemic treatment regimen in Asia, mainly in China. Studies have shown that Asian patients seem to benefit more from this regimen [10].

Thymidylate synthase (TYMS) maintains the dTMP pool, a necessary element for DNA synthesis [11, 12]. Therefore, TYMS abnormal expression is associated with a variety of tumors and is frequently used as a target for anti-tumor drugs, including 5-FU [13]. Studies revealed that TYMS serves as a biomarker for colon cancer and breast cancers, and its overexpression promotes the growth, invasion, and metastasis. Its dysregulation is closely related to 5-FU resistance [13]. In addition, overexpression of TYMS is closely associated with an unfavorable prognosis for colon cancer [14–16], breast cancer [17], pancreatic cancer [18], cervical cancer [19], non-small cell lung cancer [20], and HCC [21] patients. As for the relationship between TYMS and EMT, Siddiqui et al. reported that compared with epithelioid cancer cells, the expression of TYMS in mesenchymal cancer cells was significantly upregulated, and TYMS and ZEB1 formed a positive feedback regulation to promote the occurrence of EMT [22]. They found that the expression levels of Vimentin and ZEB1 were significantly decreased in TYMS knockdown human non-small cell lung cancer cell line A549 compared with the control cells. In contrast, the expression level of E-cadherin was increased considerably, which was similar to that observed in our study using HCC cell lines. Ahn et al.'s study also showed that overexpression of HSP90 or sustained activation of Src could upregulate the expression of TYMS in 5-FU-resistant human colon cancer cell line HCT116. The overexpression of TYMS was correlated with the decrease of E-cadherin [23]. Few studies explored the specific mechanisms of TYMS regulating the EMT process. Shi et al. reported that in breast cancer cells, TYMS promoted EMT through the HIF1alpha-CXCL12-ACKR3 signaling pathway, PLK1 or SMAD5 [24]. TYMS has been poorly studied in HCC. In this study, we explored the expression and function of TYMS in HCC and also investigated the effects of TYMS on EMT in HCC. By analyzing the TCGA LIHC dataset and using the qRT-PCR to detect the mRNA expression levels of TYMS, we found that TYMS is highly expressed in HCC samples and cell lines. We also found that TYMS overexpression promotes the growth of HCC cells. TYMS overexpression also accelerated the EMT in HCC cells. In addition, we found a positive correlation between the expression of FOXM1 (Forkhead Box M1) and TYMS in HCC. The TYMS expression was increased when FOXM1 was overexpressed in HCC cells using a FOXM1 overexpression (FOXM1-OE) plasmid. FOXM1 overexpression resulted in HCC cells resistance to 5-FU. After repressing TYMS in FOXM1-OE cells, HCC cells regained their sensitivity to 5-FU.

In conclusion, our study confirmed that TYMS promotes the growth and development of HCC, and that TYMS may serve as a therapeutic target for advanced HCC patients.

## Materials and methods

### Patient samples and cell lines

Thirty-two HCC patients (22 males and 10 females, average age: 58.2 ± 7.3) were enrolled in the present study between March 2018 and April 2020 through the Department of Radiation Oncology of Hainan Cancer Hospital. All patients were pathologically diagnosed with HCC. Before surgery, patients who received any treatment (including chemotherapy, radiotherapy, immunotherapy or other therapies) were excluded. Tumor tissues and adjacent normal tissues (> 2 cm from tumor tissues) obtained from HCC patients were immediately stored in liquid nitrogen to prepare for RNA extraction. All patients signed the consent forms and agreed to partake in this study. The relationship between TYMS expression and clinical factors is shown in Table 1.

**Table 1:** The relationship between clinical factors and TYMS expression

Clinical variables	TYMS expression		
	High (n)	Low (n)	P-value
Gender			
Male	10	12	0.811
Female	5	5	
Age (y)			
≤ 60	7	10	0.492
> 60	8	7	
AFP (ng/ml)			
≤ 20	6	7	0.719
> 20	10	9	
HBV infection			
Negative	11	4	0.131
Positive	8	9	
Vascular invasion			
Negative	3	11	**0.004**
Positive	13	5	
Cirrhosis			
No	7	9	0.154
Yes	11	5	
TNM stage			
I–II	5	12	**0.013**
III–IV	11	4	

L-02, HepG2 and Hep3B cell lines were purchased from the China Center Type Culture Collection (CCTCC). HuH-7 and HCCLM3 cell lines were purchased from the American Type Culture Collection (ATCC). Cells were cultured in DMEM medium containing 10% fetal bovine serum (FBS, Gibco) supplemented with 100U/ml penicillin and 0.1 mg/ml streptomycin. Cells were cultured at 37 °C with 5% CO_2_.

### Public databases

Clinical information and TYMS expression data for HCC samples from the TCGA (The Cancer Genome Atlas) database were studied. In addition, the GEPIA 2 website tool (http://gepia2.cancer-pku.cn/) was used to compare the differences in TYMS expression levels between HCC patients and normal samples as well as between HCC patients at different stages. The Human Protein Atlas (https://www.proteinatlas.org/) was used to compare TYMS expression from the immunohistochemical perspective.

### Gene manipulation studies

To repress TYMS knockdown, shRNAs targeting TYMS (shTYMS) were generated (GenePharma (Shanghai, China)). First, a recombinant lentivirus plasmid was constructed by introducing shTYMS or scramble shRNAs into the lentivirus plasmid PLKO.1-puro. Subsequently, the recombinant plasmid was co-transfected with VSV-G into HEK-293T cells using Lipofectamine2000 for 48 h. Next, 1 ml of lentivirus supernatant containing shTYMS was added to HCC (10^4^) cells containing 5ug/ml polybrene. After 24 h, puromycin was added for the antibiotic selection and TYMS knockdown cell lines were obtained.

For FOXM1 overexpression, a FOXM1 plasmid was purchased from Addgene (#68810, pCW57.1-FOXM1c). The FOXM1 overexpression plasmid (FOXM1-OE) was transfected to HCC cells using Lipofectamine 2000 (Invitrogen, US) according to the manufacturer's instructions.

### Cell proliferation

Cell proliferation was measured using the Cell Counting Kit-8 (CCK-8) (Dojindo, Japan) assay. Cells (10^5^) were seeded into the 96-well plates and cultured for 24, 48, 72, and 96 h before subjecting them to a CCK-8 assay. Before detecting absorbance (490 nm), 10 µL CCK-8 solution was added to each well. The absorbance was measured using a microplate reader every 30 min for 3 h.

BrdU assay was conducted using BrdU cell proliferation kit (Cell Signaling Technology, US). Briefly, 10^5^ cells/well were seeded into a 96-well plate and incubated with BrdU labeling medium for 4 h in the incubator. Cells were then fixed with 4% paraformaldehyde for 30 min and incubated with anti-BrdU for 1 h at room temperature. Finally, cells were observed under a fluorescence microscope (Olympus, Japan). CFSE assay was carried out using CellTrace CFSE Cell Proliferation kit (Invitrogen, US) as instructed. Briefly, 10^6^ cells were seed into a 6-well plate and incubated with 2uM CFSE for 30 min. After washing, cells were incubated for 48 h and then detected by flow cytometry.

### Cell apoptosis

Apoptosis was detected using the Annexin V/Dead Cell kit (Invitrogen, US), according to the manufacturer's instructions. Briefly, 3*10^5^ cells were cultured for 48 h and harvested by centrifuging. After being washed with PBS, cells were incubated with 2.5 µL Annexin V, 2.5 µL Propidium iodide (PI), and 95 µL annexin-binding buffer for 15 min at room temperature in the dark. Finally, 300 ul/tube annexin-binding buffer was added to terminate the reaction, and a flow cytometer (Celesta, BD biosciences, US) was used to detect cell apoptosis. Results were analyzed using Flowjo software (TreeStar, Version 10.4). TUNEL assay was conducted using the TUNEL detection kit (Roche, CHE) as instructed. Cells were observed under a fluorescence microscope (Olympus, Japan).

### Cell invasion assay

The Transwell invasion assay was performed to analyze cell invasion. The assay was performed using a 24-well Transwell plate (8 µm pore; Corning, US). In the upper chamber, the upper surface of the filter was coated with Matrigel (BD, US) to form a gel bed. A total of 3*10^4^ cells in the serum-free medium were seeded into the upper chamber. The lower chamber was filled with medium containing 10% FBS. Cells were then cultured for 24 h. Then, cells that were on the upper surface of the chamber and the Matrigel were removed. Cells that spread to the lower surface were fixed with 1% formaldehyde solution and stained with 0.1% crystal violet. Stained cells were photographed and counted using a microscope (Olympus, Japan).

### Cell cycle

Cell cycle assays were performed using PI. In brief, 3*10^5^ cells were harvested and fixed with 70% cold ethanol. Cells were then washed with PBS and stained with PI for 30 min at 4 °C in the dark. Cell cycle was analyzed using a flow cytometer immediately after staining. Results were analyzed using ModFit LT software (Verity Software House, version 3.1).

### Immunoblotting

Protein was extracted using RIPA buffer (Pierce, US) supplemented with Protease Inhibitor Cocktail (Roche, CHE). According to the manufacturer's instructions, the protein concentration was measured using the BCA Protein Assay Kit (Abcam, US). First, 25–35 µg protein was loaded into 4–12% tris–glycine gels (Novex, US) and was separated using SDS-PAGE. Protein was then transferred to a Polyvinylidene Fluoride (PVDF) membrane at 250 mAh for 2.5 h. After blocking the membrane with 5% non-fat milk for 1 h, the membrane was incubated with primary antibodies at 4 °C overnight. The following day, the membrane was incubated with HRP-conjugated secondary antibodies 30 min at room temperature. ECL reagents and the iBright CL750 imager (Invitrogen, US) were used to image the blots.

### Immunohistochemistry

Liver samples were fixed in 10% formalin and paraffin-embedded. Tissues were cut into 8um sections and subjected to immunohistochemistry analysis. Immunohistochemistry analysis was performed following the standard protocol. In brief, sections were deparaffinized and the antigen retrieval were performed using microwave. After incubation with primary and secondary antibodies, chromogen development were carried out using the DAKO Envision System.

### Real-time PCR analysis

RNA was extracted using TRIzol solution (Invitrogen, US) according to the manufacturer's instructions. RNA was reverse transcribed to cDNA using Hiscript III Reverse Transcriptase (Vazyme, China). Then, qRT-PCR was performed using an SYBR Green PCR Master Mix from Takara. The thermal cycling parameters were as follows: Hold: 95 °C for 30 s, one cycle; 2 Step PCR: 95 °C for 5 s and 60 °C for 30 s, 40 cycles. Relative gene expression was calculated using the 2^−△△Ct^ method normalized to GAPDH. Primers for included: FOXM1 forward: 5′-AGAGCTTGCCCGCCATAG-3', reserve: 5′-CCTCCTTGATAGTCTGAACTGGA’. TYMS forward: 5′-GCATTTTGGGGCAGAATACA-3′, reserve: 5′-GGACAGCTCACTGTTCACCA-3′. GAPDH forward: 5′-GAGCCACATCGCTCAGACA-3′, and reverse: 5′-CATGTAGTTGAGGTCAATGAAGG-3′.

### Statistical analysis

All data were presented as mean ± SD. The student's t-test was used for comparison between 2 groups, while one-way ANOVA was used to compare multiple groups. Bonferroni test was used as the post-hoc test. The P-value was calculated using SPSS software (IBM, 22.0 version), and P < 0.05 was considered statistically significant. Kaplan–Meier curves were used to analyze survival. All experiments were conducted as three independent replicates.

## Results

### TYMS was upregulated in HCC tissues and cell lines

**Fig. 1:**
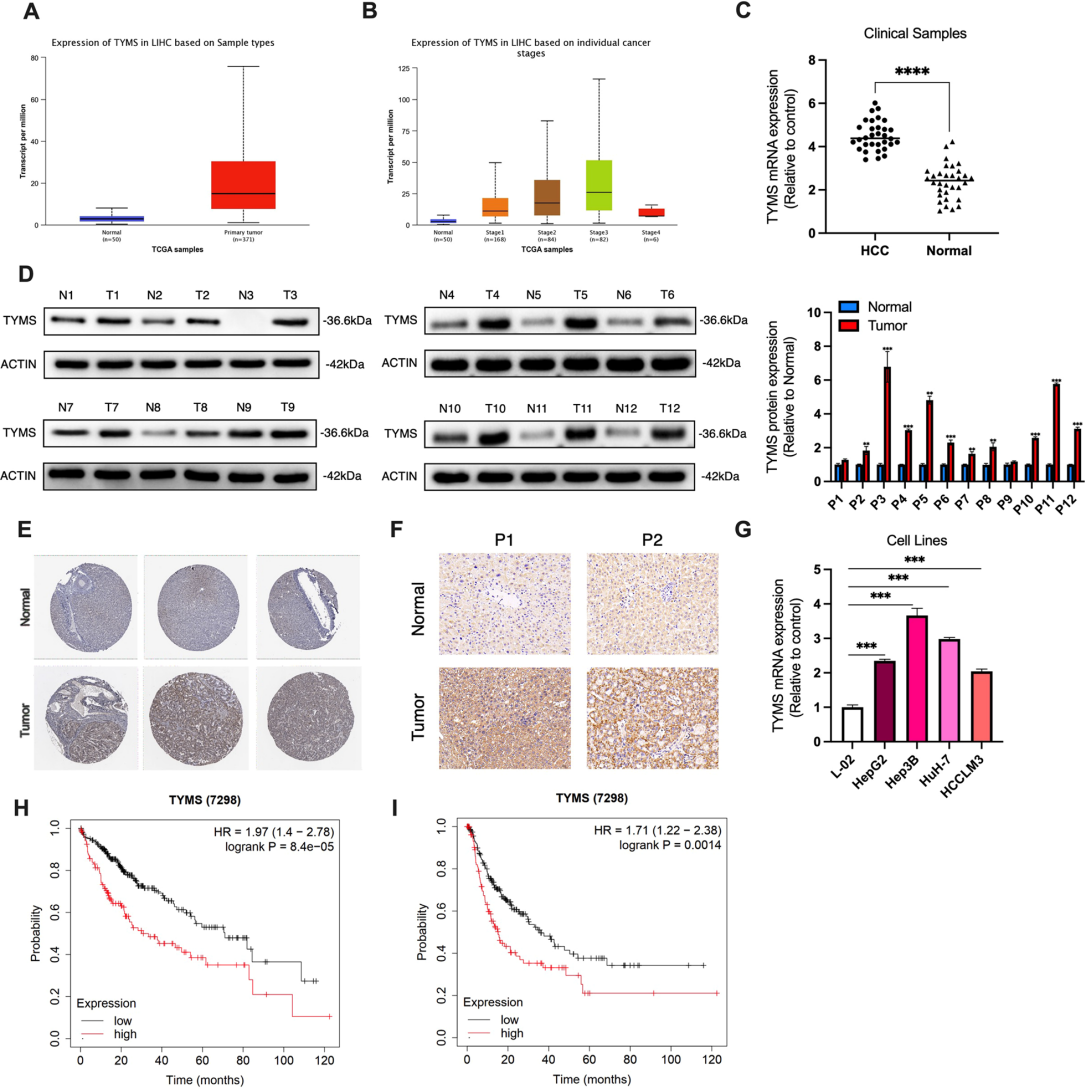
TYMS was highly expressed in HCC samples and was associated with shortened OS and PFS. **A** The expression level of TYMS in tumor (n = 371) and normal tissues (n = 50) in the TCGA-LIHC dataset. **B** The expression level of TYMS in HCC tissues of different TNM stages. **C** The mRNA expression level of TYMS in collected clinical samples (n = 32). **D** The protein expression of TYMS in normal liver tissues and HCC tissues from clinical samples. **E** Immunohistochemical images of TYMS from The Human Protein Atlas in normal liver and HCC tissues. **F** Immunohistochemistry of TYMS in HCC tissues and adjacent normal tissues in 2 patients. **G** The expression level of TYMS in human normal liver cell line L-02 and several HCC cell lines. The relationship between TYMS expression and **H** OS and **I** PFS in the TCGA-LIHC dataset. *P < 0.05, **P < 0.01, ***P < 0.001, ****P < 0.0001

We first explored the expression of TYMS in HCC samples using the TCGA LIHC dataset. Using the GEPIA website tool, we found that the mRNA expression level of TYMS was significantly increased in HCC tissues compared with the normal controls, and its expression was gradually increased with the progression of HCC (in the first three stages) (Fig. 1A, B). However, we did not observe a significant increase in TYMS at Stage 4, possibly due to the small sample size for this stage (Fig. 1B). To confirm this finding, we examined TYMS expression levels in clinical samples. As shown in Fig. 1C and D, our result were consistent with the TCGA dataset (Fig. 1C). Immunohistochemical data from The Human Protein Atlas also suggested that TYMS expression in HCC tissues was significantly higher than in normal liver tissues (Fig. 1E). TYMS expression was further observed in cell lines. These results showed that TYMS was upregulated in HCC cell lines compared to L-02, a human normal liver cell line (Fig. 1F). We then sought to determine whether TYMS affected the prognosis of HCC. Survival analysis suggested that higher TYMS expression was associated with both unfavorable overall survival (OS) (Fig. 1G) and progress-free survival (PFS) (Fig. 1H). Upregulation of TYMS was related to positive vascular invasion and advanced TNM stages in collected samples (Table 1).

### TYMS knockdown suppressed the proliferation and enhanced the apoptosis of HCC cells

**Fig. 2:**
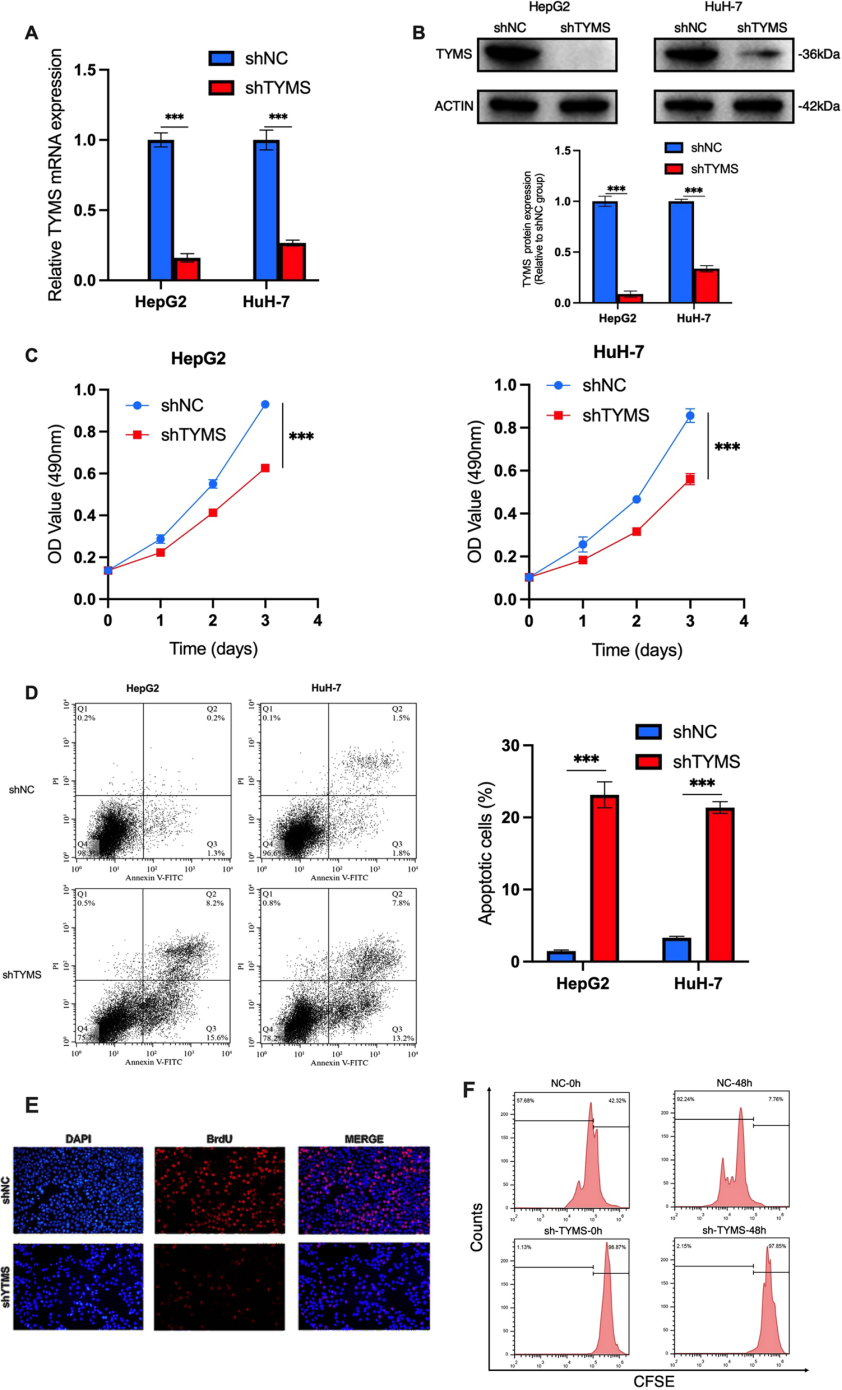
TYMS knockdown inhibited HCC cell proliferation and promoted apoptosis. TYMS **A** mRNA and **B** protein expression level in two HCC cell lines HepG2 and Huh-7 after TYMS knockdown. **C** CCK-8 and **D** flow cytometry apoptosis assay was performed in shTYMS and NC HCC cells to detect TYMS effects on cell growth. **E** BrdU assay and **F** CFSE assay in shNC or shTYMS HepG2 cells confirmed that TYMS knockdown inhibited HCC cell proliferation.*P < 0.05, **P < 0.01, ***P < 0.001, ****P < 0.0001

We explored the role of TYMS in the HCC cell lines HepG2 and HuH-7. TYMS was silenced using shTYMS through lentivirus transfection. RT-qPCR and western blot analysis confirmed that shTYMS successfully repressed TYMS expression (Fig. 2A, B). CCK-8 and apoptosis assays were performed using shTYMS and shNC (scramble shRNA) transfected cells. The results showed that silencing TYMS expression suppressed the proliferation and increased the apoptosis of both HepG2 and HuH-7 cell lines (Fig. 2C, D) significantly. For cell proliferation, we further performed CFSE and BrdU assay in HepG2 cells to confirm our findings. As shown in Fig. 2E, F, TYMS knockdown significantly inhibited cell proliferation in HepG2 cells. These findings were similar to that in CCK-8 assays. For cell apoptosis, we also conducted the TUNEL assay and western blot assay for caspase 3 and 7 in HepG2 cells to verify the promoting effect of TYMS knockdown on HCC cell apoptosis. As shown in Additional file 2: Fig. S2, TYMS knockdown considerably increased the apoptosis rate of HepG2 cells. The results were similar to those in the AnnexinV apoptosis assay.

**Fig. 3:**
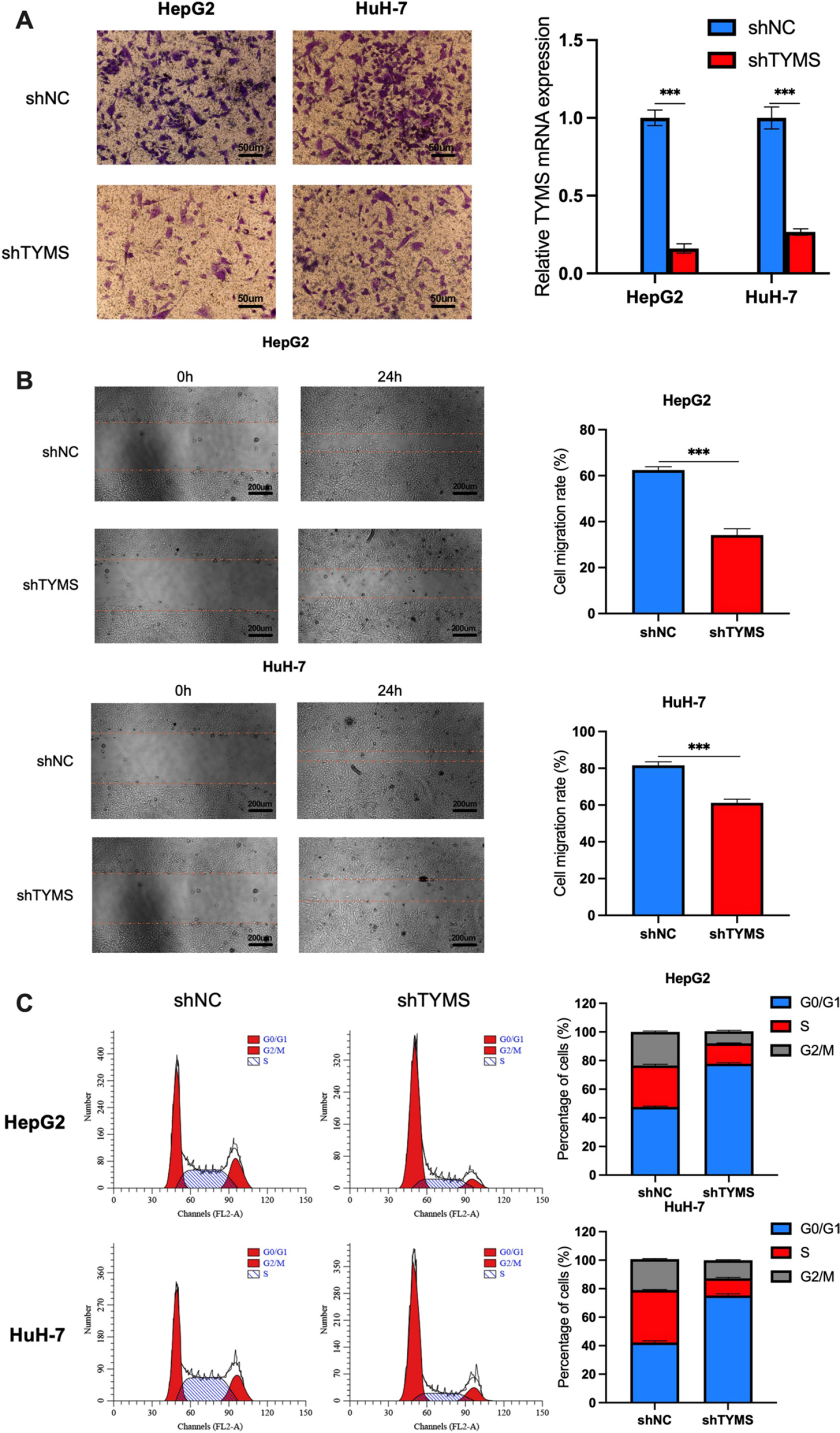
TYMS knockdown suppressed cell invasion, migration and arrested cell cycle at G0/G1 phase. **A** Transwell invasion assays, **B** wound healing assays and **C** cell cycle assays in HepG2 and HuH-7 cells with/without TYMS knockdown. *P < 0.05, **P < 0.01, ***P < 0.001, ****P < 0.0001

We next performed Transwell invasion assay, wound healing assay, and flow cytometry cell cycle assays to investigate the effects of TYMS knockdown on cell invasion and the cell cycle. As shown in Fig. 3A, the number of invasive cells significantly decreased after TYMS knockdown. Consistent with this finding, the wound healing assay also showed a considerable reduction in cell migration rate (Fig. 3B). In addition, TYMS knockdown raised the percentage of cells in the G0/G1 phase for the cell cycle, indicating that TYMS silencing leads to G0/G1 phase in HCC cells (Fig. 3C).

### TYMS knockdown suppressed the epithelial-mesenchymal transition (EMT) in HCC cells

**Fig. 4:**
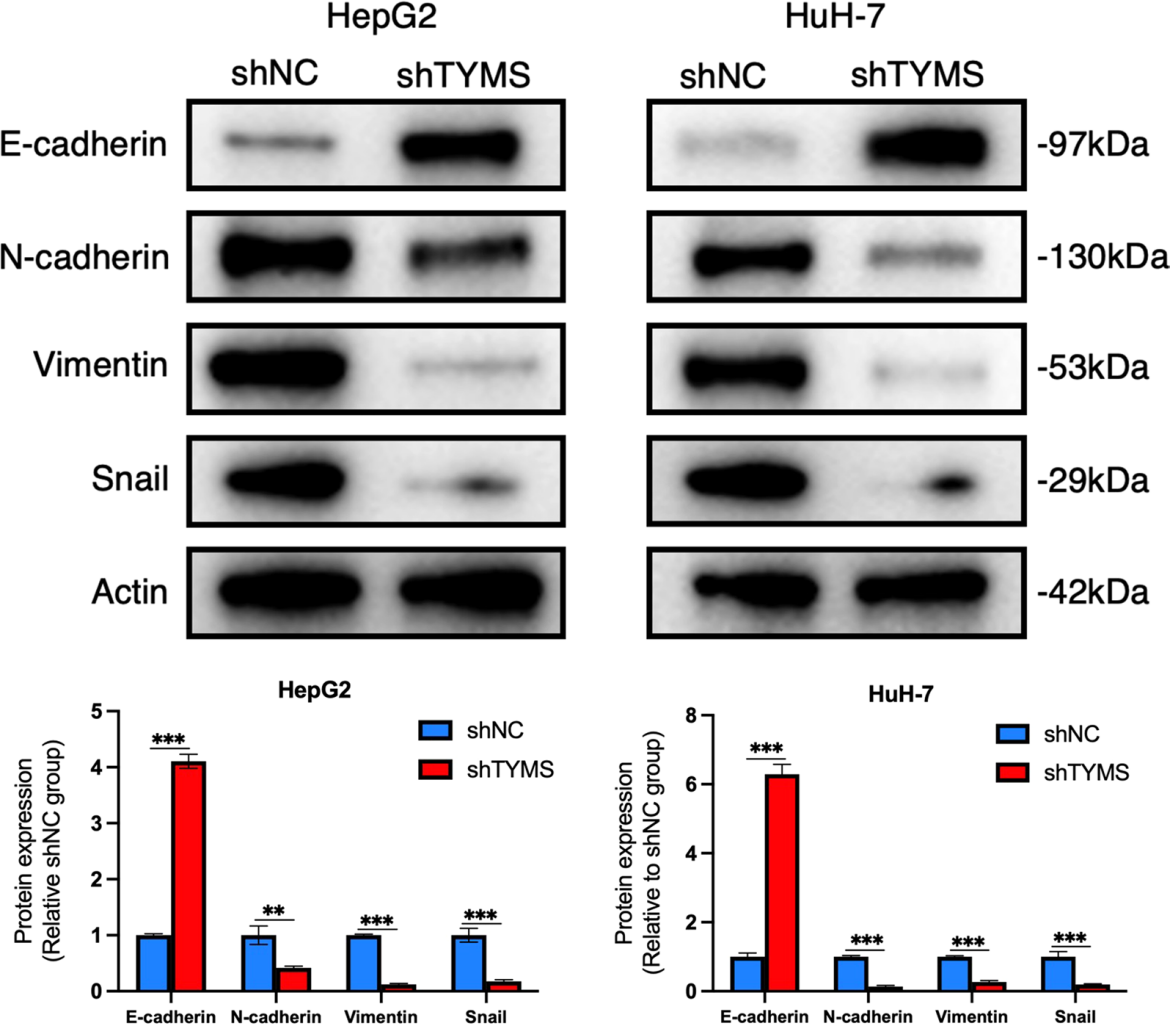
TYMS knockdown inhibited EMT in HCC. Protein expression of EMT related-genes detected by western blot assays in HepG2 and HuH-7 cells with/without TYMS knockdown. *P < 0.05, **P < 0.01, ***P < 0.001, ****P < 0.0001

EMT is closely related to cell invasion and tumor progression. To explore the mechanisms behind reduced cell invasion in shTYMS HCC cells, we performed a western blot assay in cell lines with repressed TYMS to examine the expression of EMT-related proteins. The results revealed that the expression of E-cadherin was significantly upregulated after TYMS knockdown in both HepG2 and HuH-7 cells. In contrast, the expression levels of N-cadherin, Vimentin, and Snail showed the opposite changes (Fig. 4). These data suggested that TYMS silencing impeded EMT in HCC cells.

### FOXM1 regulated TYMS expression

**Fig. 5:**
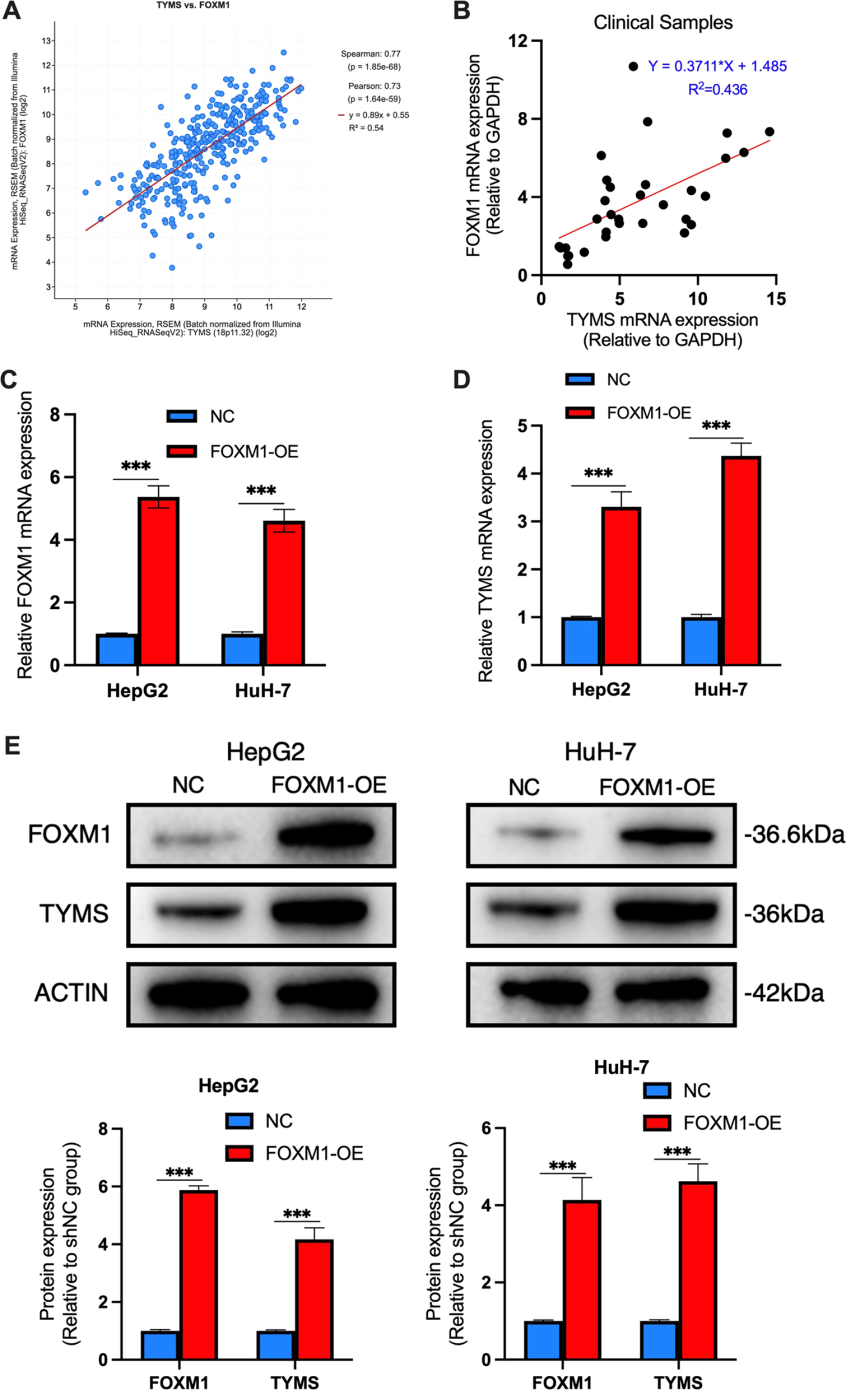
TYMS was a downstream target of FOXM1. The linear relationship between FOXM1 and TYMS in **A** the TCGA-LIHC dataset and **B** the collected samples. The expression level of **C** FOXM1 and **D** TYMS mRNA in HepG2 and Huh-7 cells with/without transfection of FOXM1-OE plasmid. **E** The expression level of FOXM1 and TYMS protein in HepG2 and Huh-7 cells with/without transfection of FOXM1-OE plasmid. *P < 0.05, **P < 0.01, ***P < 0.001, ****P < 0.0001

We next explored the regulatory mechanisms of TYMS in HCC cells. The cBioPortal website tool was used to analyze genes that are significantly associated with the expression of TYMS, one of these genes being FOXM1 (Fig. 5A). We found that FOXM1 was highly correlated with tumor EMT, and previous studies suggested that FOXM1 regulated the expression of TYMS and mediated the resistance of tumor cells to 5-FU in colon cancer [15]. We also explored the relationship between FOXM1 and TYMS in primary samples to find similar results (Fig. 2B). To further investigate whether FOXM1 regulated TYMS, we transfected a FOXM1 overexpression (FOXM1-OE) plasmid into HCC cells to construct FOXM1-overexpressing HCC cell lines. The qRT-PCR results showed that the mRNA expression of FOXM1 in the FOXM1-OE HepG2 and HuH-7 cells was significantly higher than that in the NC group (Fig. 5C). In addition, the expression of TYMS in FOXM1-OE and NC cells was detected. The results showed that the expression of TYMS was significantly upregulated at both mRNA and protein levels in FOXM1-OE cells (Fig. 5D, E).

### Overexpression of FOXM1 neutralizes the tumor inhibition effects induced by TYMS knockdown and mediates the resistance to 5-FU in HCC cells

**Fig. 6:**
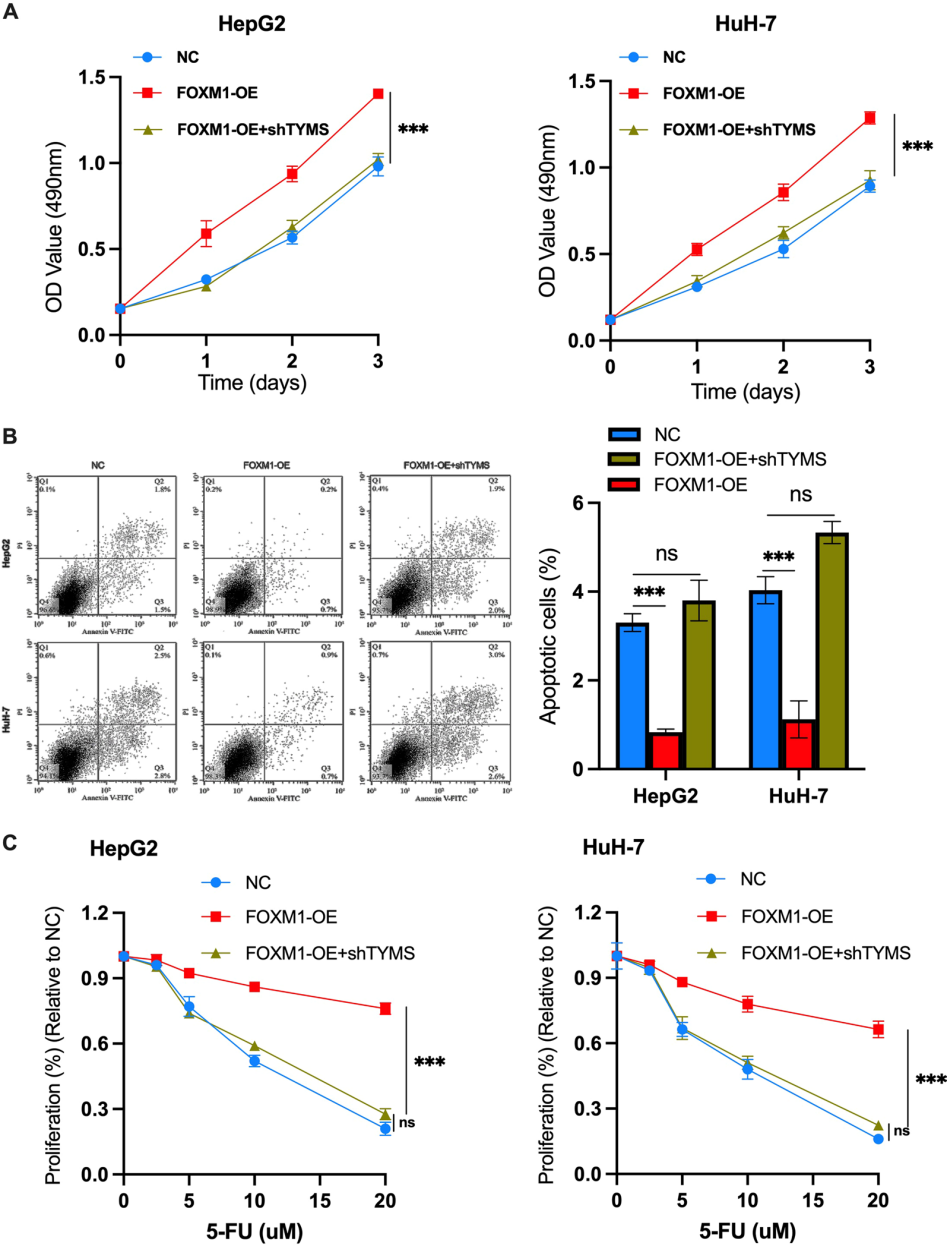
The FOXM1-TYMS axis regulated the growth of HCC cells and mediated the resistance of cells to 5-FU. **A** CCK-8 assays, **B** flow cytometry apoptosis assays in HepG2 and HuH-7 cells transfected with FOXM1-OE or FOXM1-OE plasmid + shTYMS or negative control. **C** CCK-8 assays in HepG2 and HuH-7 cells transfected with FOXM1-OE plasmid or FOXM1-OE plasmid + shTYMS or negative control. All cells were treated with 5-FU at indicated concentrations for 24 h. *P < 0.05, **P < 0.01, ***P < 0.001, ****P < 0.0001

Next, we investigated whether the FOXM1-TYMS axis played a vital role in the progression of HCC. After FOXM1-OE and shTYMS were co-transfected into HCC cells, CCK-8 and cell apoptosis assays were performed. For the CCK-8 assay, no significant difference was observed in cell proliferation rate for NC and FOXM1-OE + shTYMS HCC cells, but cell proliferation ability was enhanced in FOXM1-OE cells (Fig. 6A). Apoptosis of FOXM1-OE cells was significantly reduced compared with NC cells (Fig. 6B). We also investigated the effects of FOXM1 on 5-FU killing HCC cells. As shown in Fig. 6C, FOXM1 overexpression increased HCC cells resistance to 5-FU, and knockdown of TYMS alleviated FOXM1-induced drug resistance. In addition, CCK-8 assays showed that TYMS knockdown failed to overcome sorafenib resistance caused by FOXM1 overexpression (Additional file 1: Fig. S1). Combined, these findings suggested that the FOXM1-TYMS axis has significant regulatory effects on the survival of HCC cells and mediates HCC cells' resistance to 5-FU based chemotherapy.

## Discussion

Treatment of advanced HCC has always been challenging [25]. This is mainly a result of lymph node and distant metastases in advanced HCC. Even after radical surgical treatment, there is an excessive probability for recurrence. Therefore, it is necessary to explore the mechanisms of HCC metastasis to better understand how to treat advanced stages.

TYMS is a gene that plays a crucial role in DNA repair and replication. It catalyzes the synthesis of dTMP, a necessary precursor of DNA synthesis, and is one of the targets of anti-tumor drugs such as 5-FU or TDX [26, 27]. In addition, it is involved in the progression and drug resistance of advanced breast cancer [28]. It is abnormally expressed in melanoma and is thought to be associated with the prognosis of early colon cancer [29] and postoperative small cell lung cancer [30]. In addition, TYMS is associated with tumor metastasis and aggressive phenotypes of several tumors, such as glioma [31], breast cancer [32], prostate cancer [33], and soft tissue sarcoma [34]. However, little is known about TYMS in HCC. Although a previous study suggested that high expression of TYMS may be an independent prognostic factor for HCC, their results are not statistically significant [35]. In this study, we found that high expression of TYMS was significantly associated with poor OS and PFS using the data extracted from the TCGA-LIHC dataset. Meanwhile, in vitro experiments demonstrated that TYMS knockdown inhibited HCC cell proliferation, enhanced cell apoptosis, and suppressed cell migration and EMT.

FOXM1, a transcriptional factor associated with cell proliferation, was associated with enhanced cell proliferation, metastasis, and EMT in HCC [36–38]. Hu et al. reported that KIF4A is a direct target of FOXM1. FOXM1-induced HCC cell proliferation is mediated by KIF4A upregulation [39]. Shang et al. found that FOXM1 promotes HCC progression through trans-activating GLUT1 [40]. Xia et al. suggested that FOXM1 accelerates HCC invasion and metastasis through upregulating MMP-7, RhoC and ROCK1 [41]. Besides, previous studies revealed that as a transcriptional factor, FOXM1 regulates HCC progression through regulating or interacting with a number of target genes, including but not limited to Aurora Kinase A, Cdc2, Nek2 [42], TPX2 [43], CCNB1 [36], LINC-ROR [42] and UHMK1 [44]. Besides, TYMS may be a downstream target of FOXM1 [15]. Upregulation of FOXM1 was suggested to be associated with sorafenib resistance in HCC [45]. In this study, we also found that TYMS expression was positively correlated with the expression of FOXM1. Furthermore, we found that TYMS mRNA and protein levels increased after overexpressing FOXM1 in HCC cells. These results confirmed that TYMS was one of the target genes of FOXM1 in HCC. Next, we explored whether 5-FU resistance in HCC was affected by the FOXM1-TYMS axis. Results indicated that cell proliferation was significantly increased, and apoptosis was significantly decreased in FOXM1-OE HCC cells, and the cells were resistant to 5-FU. Similar results were found in colorectal cancer [15]. However, after co-transfection of FOXM1-OE cells with shTYMS, the oncogenic effects of FOXM1 on cell growth, survival, and 5-FU resistance were attenuated. We also conducted CCK-8 assays using sorafenib instead of 5-FU, and the results suggested that although FOXM1 upregulation induced sorafenib resistance in HCC cells, this effect was not mediated through the FOXM1-TYMS axis (Additional file 1: Fig. S1).

Previous studies have shown that FOXM1-TYMS mediates 5-FU resistance of tumor cells in colon cancer [15]. However, there have been no similar studies in hepatocellular carcinoma. Because in different cancer and genetic background, the function and expression of the same gene are not the same. For example, KLF4 has multiple functions and can be either a tumor suppressor gene or a proto-oncogene in tumors. KLF4 plays a tumor suppressor role in gastrointestinal tumors [46] but it promotes HCC progression [47]. For TYMS in HCC, one possibility is that although TYMS mediates 5-FU resistance in colon cancer, it means nothing in HCC. Therefore, it is necessary to explore its function in hepatocellular carcinoma.

In conclusion, this study suggested TYMS serves as an oncogene in HCC and that targeting the FOXM1-TYMS axis could help improve survival and provide new ideas for treating patients with advanced HCC.

## Supplementary Information

**Additional file 1: Figure S1.** The FOXM1 induced sorafenib resistance in HCC cells, but this effect was not achieved through TYMS. CCK-8 assays in (A) HepG2 and (B) HuH-7 cells transfected with FOXM1-OE plasmid or FOXM1-OE plasmid + shTYMS or negative control. All cells were treated with sorafenib at indicated concentrations for 24 h.

**Additional file 2: Figure S2.** TYMS knockdown increased HCC cell apoptosis. (A) TUNEL and (B) Western Blot analysis for caspase 3 and 7 in shNC or shTYMS HepG2 cells.

## Data Availability

All data generated or analyzed during this study are included in this published article and its supplementary information files.
