# Correction: FOXM1-induced TYMS upregulation promotes the progression of hepatocellular carcinoma

**DOI:** 10.1186/s12935-022-02802-9

**Published:** 2022-12-07

**Authors:** Liang Wang, Caiyan Shi, Jie Yu, Yilin Xu

**Affiliations:** 1 The Department of Radiation Oncology, Hainan Cancer Hospital, Haikou, 570311 Hainan China; 2 The Department of Medical Oncology, Hainan West Central Hospital, Danzhou, 571700 Hainan China; 3 Department of Anal-Colorectal, Jingzhou Central Hospital, The Second Clinical Medical College, Yangtze University, Jingzhou, 434020 Hubei China; 4 Department of Nephrology, Affiliated Huaian Hospital of Xuzhou Medical University, Huaian, 223001 Jiangsu China

**Correction: Cancer Cell International (2022) 22: 47** https://doi.org/10.1186/s12935-021-02372-2

In this article [1], the affiliation details of the authors Dr. Caiyan Shi and Yilin Xu was transposed. It has been corrected with this erratum.

The correct affiliations are given below:

Caiyan Shi: The Department of Medical Oncology, Hainan West Central Hospital, Danzhou, 571700, Hainan, China.

Yilin Xu: Department of Nephrology, Affiliated Huaian Hospital of Xuzhou Medical University, Huaian, 223001, Jiangsu, China.
