# Supplementary figures and images for: FOXM1-induced TYMS upregulation promotes the progression of hepatocellular carcinoma
Source: Cancer Cell Int. 2022 Jan 29;22:47. doi: 10.1186/s12935-021-02372-2 (PMC8801073; doi:10.1186/s12935-021-02372-2)

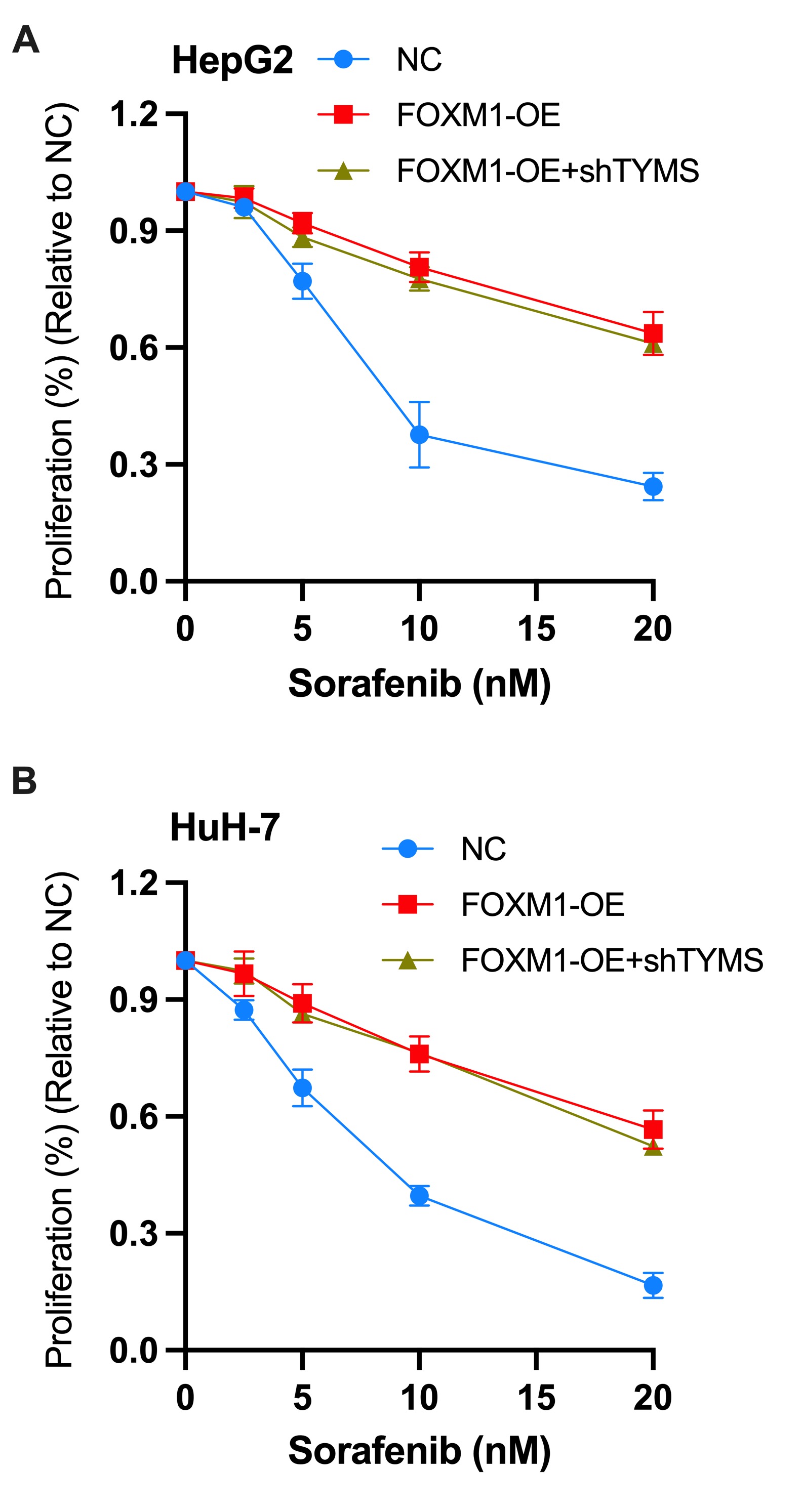

Supplement: Supplementary file 1 — Additional file 1: Figure S1. The FOXM1 induced sorafenib resistance in HCC cells, but this effect was not achieved through TYMS. CCK-8 assays in (A) HepG2 and (B) HuH-7 cells transfected with FOXM1-OE plasmid or FOXM1-OE plasmid + shTYMS or negative control. All cells were treated with sorafenib at indicated concentrations for 24 h. [file 12935_2021_2372_MOESM1_ESM.jpg]

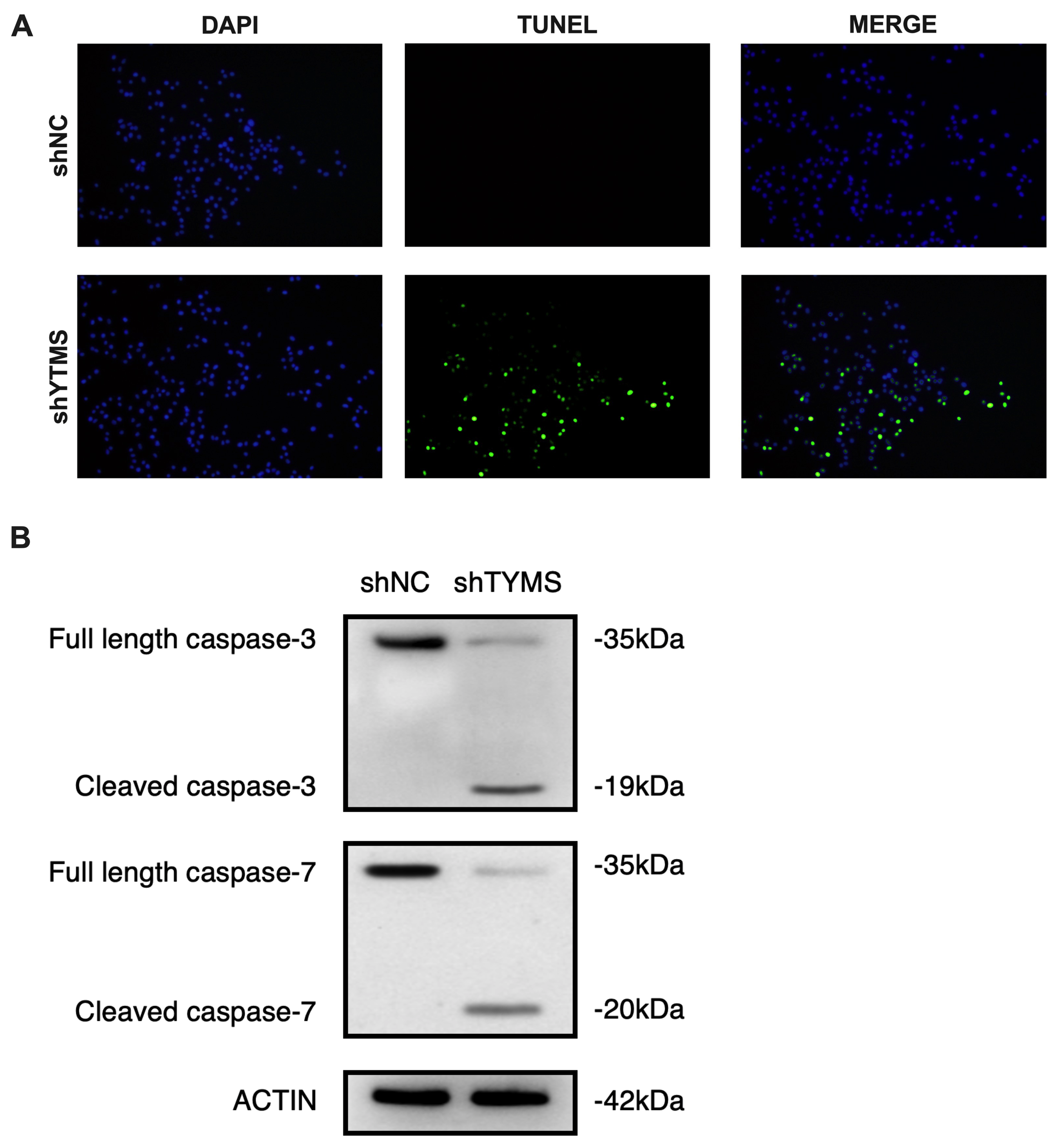

Supplement: Supplementary file 2 — Additional file 2: Figure S2. TYMS knockdown increased HCC cell apoptosis. (A) TUNEL and (B) Western Blot analysis for caspase 3 and 7 in shNC or shTYMS HepG2 cells. [file 12935_2021_2372_MOESM2_ESM.tiff]
